# Emerging roles of RNA methylation in gastrointestinal cancers

**DOI:** 10.1186/s12935-020-01679-w

**Published:** 2020-12-07

**Authors:** Shanshan Xie, Wenwen Chen, Kanghua Chen, Yongxia Chang, Feng Yang, Aifu Lin, Qiang Shu, Tianhua Zhou, Xiaoyi Yan

**Affiliations:** 1 The Children’s Hospital, Zhejiang University School of Medicine, Hangzhou, 310052 China; 2 Department of Cell Biology, Zhejiang University School of Medicine, Hangzhou, 310058 China; 3 MOE Laboratory of Biosystem Homeostasis and Protection, College of Life Sciences, Zhejiang University, Hangzhou, 310058 China; 4 Department of Molecular Genetics, University of Toronto, Toronto, ON Canada

**Keywords:** RNA methylation, m6A, m6Am, m1A, m5C, Gastrointestinal cancers

## Abstract

RNA methylation has emerged as a fundamental process in epigenetic regulation. Accumulating evidences indicate that RNA methylation is essential for many biological functions, and its dysregulation is associated with human cancer progression, particularly in gastrointestinal cancers. RNA methylation has a variety of biological properties, including *N*6-methyladenosine (m6A), 2-*O*-dimethyladenosine (m6Am), *N*1-methyladenosine (m1A), 5-methylcytosine (m5C) and 7-methyl guanosine (m7G). Dynamic and reversible methylation on RNA is mediated by RNA modifying proteins called “writers” (methyltransferases) and “erasers” (demethylases). “Readers” (modified RNA binding proteins) recognize and bind to RNA methylation sites, which influence the splicing, stability or translation of modified RNAs. Herein, we summarize the biological functions and mechanisms of these well-known RNA methylations, especially focusing on the roles of m6A in gastrointestinal cancer development.

## Background

Gastrointestinal (GI) cancers refer to malignant conditions of the GI tract and accessory organs of digestion including liver and pancreas. Globally, GI cancers account for roughly half of all cancer-related deaths [1, 2]. GI cancers have high mortality rates, mainly because of asymptomatic at early-stages, and limited treatment options and poor prognosis at advanced stages. Thus, the identification of robust biomarkers for early-stage and the development of new drugs to treat GI cancers are urgently needed.

Even though modification events in RNA molecules were discovered in the 1950s, our understanding of RNA modification is limited [3–6]. Recently, with the rapid development of transcriptomics technologies, studies about the physiological and pathological function of RNA modification come to the forefront. To date, over 170 RNA modifications have been identified, including RNA methylation and pseudouridylation (Ψ). These modifications have been identified to distribute extensively in kinds of RNAs, such as messenger RNAs (mRNAs), ribosomal RNAs (rRNAs), transfer RNAs (tRNAs), small nuclear RNAs (snRNAs) and small nucleolar RNAs (snoRNAs) [7].

Among RNA modifications, RNA methylation is the most well-characterized type. Highly dynamic and reversible methylation on RNA is mediated by a number of proteins, which are called RNA-modifying proteins (RMPs) [8, 9]. RMPs include “writers” and “erasers” that respectively decorate and remove methylations on RNA respectively, and ‘‘readers’’ that recognize and bind to the methylation sites. The fates of modified RNA depends on the functions of distinct “readers” that may affect their metabolism process (splicing, stability or translation). RNA methylation plays critical roles in diverse physiological processes, and its alterations has been linked to various human cancer [10–16].

In this review, we focus on metabolic regulatory mechanisms and biological functions of RNA methylation. Moreover, we summarize the role of dysregulated m6A modifying proteins in cancer initiation and progression, and discuss the potential of targeting m6A modifying proteins with aberrant expression for therapy.

## RNA methylation

**Fig. 1:**
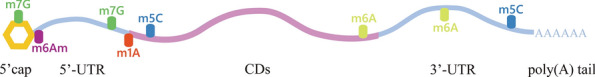
The distribution of methylation in mRNA. The preferential locations of each methylation within mRNA are shown. *m6A* *N*6-methyladenosine, *m6Am* 2-*O*-dimethyladenosine, *m1A* *N*1-methyladenosine, *m5C* 5-methylcytosine, *m7G* 7-methyl guanosine

In chemistry, methylation means a form of alkylation that adds a methyl group on a substrate or substitutes the original atom or group [17]. In biological systems, methylation confers to epigenetic alterations to regulate its expression but not affect gene sequence. It can occur in varieties of biomolecules including DNA, RNA and proteins. RNA methylation was first reported as early as the 1950s. Gutman et al. found that *N*6-methyladenine, *N*2-methylguanine and 1-methylguanine occurred in the ribonucleic acid hydrolysates in yeast [18]. To date, more than 70 of RNA methylations have been found in diverse kinds of RNA molecules across species. RNA methylation is a reversible post-translational modification that epigenetically affects many biological processes, such as RNA stability, localization, mRNA translation and translocation [19, 20]. Here, we will introduce some well-known types of RNA methylation, particularly *N*6-methyladenosine (m6A), 2-*O*-dimethyladenosine (m6Am), *N*1-methyladenosine (m1A), 5-methylcytosine (m5C) and 7-methyl guanosine (m7G) (Fig. 1).

### m6A modification

**Fig. 2:**
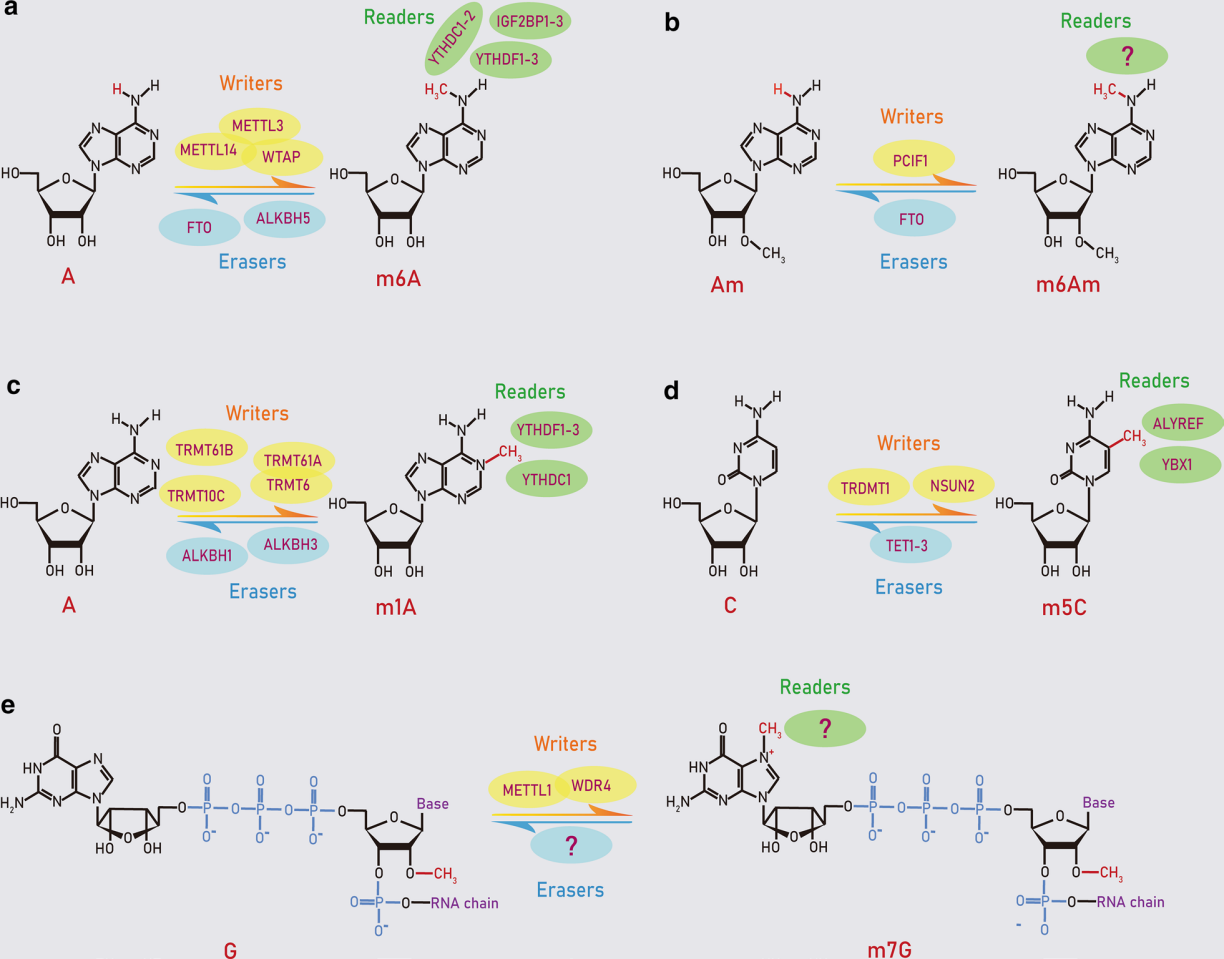
Molecular composition of RNA methylation. Reversible methylations on RNAs are mediated by RNA modifying proteins called “writers” (yellow), “erasers” (blue). “Readers” (green) are also shown

m6A refers to methylation of the adenosine base at the nitrogen-6 position (Fig. 2a). It was originally identified in mRNA in 1974. So far, m6A has been considered as the most abundant internal methylation in mRNA, with around 25% of mRNA carrying at least one m6A site [3, 21, 22]. In 2012, a transcriptome-wide m6A site mapping reveals that m6A sites are present on more than 7600 mRNAs and 300 noncoding RNAs, and are enriched near the stop codons and in the 3′ UTRs of mRNAs of human and mice. m6A is also found in rRNA, tRNA and snRNA. Almost 90% of m6A peaks contain at least one of m6A consensus motifs [23, 24]. The predominant consensus sequence is “A/G-A/G-methylated A-C-U”.

m6A is dynamically regulated by specific methyltransferases and demethylases. The most well-characterized methyltransferases is the core methyltransferase complex, which consists of methyltransferase-like 3 (METTL3) and methyltransferase-like 14 (METTL14) (Fig. 2a). METTL3, an *S*-adenosyl-l-methionine (SAM)-binding protein, is identified as a catalytic subunit in this complex, while METTL14 serves as an essential component to facilitate RNA binding [25]. Recent research shows that Wilms tumor 1-associating protein (WTAP) interacts with the METTL3–METTL14, which acts as a third subunit of the complex [26]. Fat mass and obesity-associated protein (FTO) is the first m6A eraser discovered, which catalyze the oxidation of m6A to *N*(6)-hydroxymethyladenosine (hm6A) as an intermediate modification, and further oxidize hm6A to *N*(6)-formyladenosine [27, 28]. AlkB homolog 5 (ALKBH5), another well-known demethylase, directly removes the methyl group from the methylated adenosine but not oxidative demethylation [29]. Several reader proteins directly recognize and bind m6A-containing mRNAs, including YTH-domain-containing family proteins (YTHDF1-3, YTHDC1-2) and insulin-like growth factor 2 mRNA-binding proteins (IGF2BP1-3), which is involved in RNA fate decision [12, 30, 31].

### m6Am modification

m6Am was discovered at the 5′ ends of up to 30% mRNAs in 1975 [32]. The first nucleotide after an m7G cap can be methylated on the ribose sugar to form 2′-*O*-methyladenosine (Am) (Fig. 2b). Then, Am could be further methylated at its N6 position to generate m6Am [33, 34]. A transcriptome-wide m6Am mapping reveals that the m6Am/A ratio of total RNA ranges from 0.0036 to 0.0169% in human tissues, and the m6Am levels show a negative correlation with the corresponding protein expression [35]. More recent studies identify phosphorylated CTD interacting factor 1 (PCIF1) as a mammalian mRNA m6Am methyltransferase [36–38]. In addition, FTO has been reported to convert m6Am to Am in cellular mRNAs [39, 40].

### m1A modification

m1A is a reversible methylation with the addition of a methyl group and a positive charge at the N1 position of adenosine, which blocks the Watson–Crick interface, alters RNA secondary structures and protein-RNA interactions [41] (Fig. 2c). The first study on m1A in total RNA was back to 1961 [42]. Later, m1A was found in rRNA, tRNA, mRNA and mitochondrial RNAs [43–48]. m1A was highly abundant in tRNA and rRNA, while it displayed a low abundance in mRNA. The m1A/A ratio in mammalian cells is about 0.02% and up to 0.16% of mammalian tissues [45, 46].

The best-characterized m^1^A methyltransferase responsible for cytoplasmic tRNAs has two subunits, TRMT6 and TRMT61A in eukaryote [49] (Fig. 2c). TRMT61A has tRNA adenine-*N*1-methyltransferase catalytic activity, and TRMT6 is important for tRNA binding [50]. In addition, TRMT10C and TRMT61B are known to catalyze m1A at positions 9 and 58 in mitochondria tRNAs [51, 52]. Recent studies find that these tRNA methyltransferases may also catalyze m1A in mRNAs [47, 48]. For example, TRMT6/61A writes m1A in some mRNAs carrying a GUUCRA tRNA-like motif, whereas TRMT61B and TRMT10C are able to add m1A sites in some mitochondria mRNAs. Similar to m6A, two AlkB family proteins ALKBH1 and ALKBH3 have been reported to demethylate m1A sites in RNAs [46, 53]. ALKBH1 is an m1A demethylase in cellular tRNAs [53], while ALKBH3 is identified as a demethylase in both mRNAs and tRNAs [46]. A recent study suggests that YTHDF1-3 and YTHDC1, but not YTHDF2, are putative m1A readers [54].

### m5C modification

m5C occurs in both DNA and RNA by introducing a methyl group in the 5th carbon atom of cytosine [55] (Fig. 2d). The highly abundant m5C exists in a number of RNAs, including mRNAs, tRNAs, rRNAs and enhancer RNAs (eRNAs). m5C sites in mRNA are generally located in the vicinity of the argonaute-binding regions within the 3′ UTR or near the translational start site [56, 57]. In eukaryotic, m5C is introduced by the DNA methyltransferase homolog DNMT2 or the NSUN family members (NSUN1-7) [56, 58]. NSUN2 catalyzes the m5C in mRNAs, or at position 34 of intron-containing tRNA^(Leu) (CAA)^ precursors [56, 59]. The major RNA targets of DNMT2 is tRNA^Asp^, tRNA^Gly^ and tRNA^Val^ [60]. The ten–eleven translocation (Tet) family proteins (TET1-3) are Fe(II)^−^ and 2-oxoglutarate-dependent dioxygenases to demethylate m5C sites in mammalian RNAs [61]. These TET enzymes possess the catalytic activity to induce the formation of 5-hydroxymethylcytidine in RNAs. Additionally, the mRNA export adaptor Aly/REF export factor (ALYREF, also known as THOC4) and Y-box binding protein 1 (YBX1) have been reported as an m5C readers for mRNAs, which recognizes and binds to m5C-modified mRNAs [57, 62].

### m7G modification

Most eukaryotic mRNAs have a methyl group and a positive charge at the N7 position of the terminal guanosine at its 5′ cap (Fig. 2e). m7G is present at not only mRNA caps, but also some internal positions within mRNAs, tRNAs and rRNAs [63, 64]. The most well-characterized enzyme mediating internal m7G methylation is METTL1, which works together with its co-factor WD repeat domain 4 (WDR4) [65].

## Biological functions of RNA methylation

Recently, the rapid development of transcriptome-wide profiling of diverse RNA methylations, along with the identification of the corresponding writers, erasers and readers, enable researchers to reveal the regulatory roles of RNA methylation in RNA metabolism and a wide range of cellular and biological processes.

### The roles of RNA methylations in RNA metabolism

**Fig. 3:**
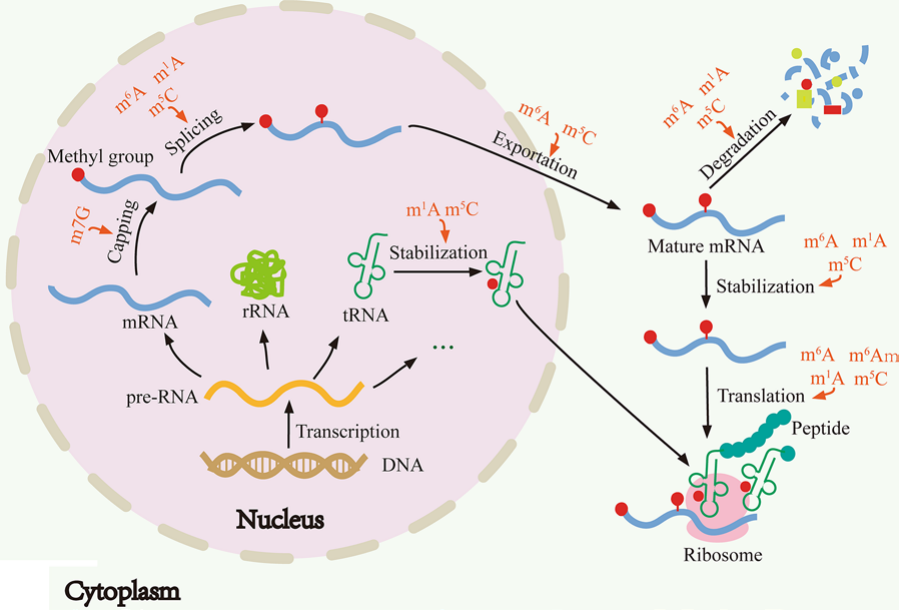
The roles of RNA methylation in RNA metabolism. RNA methylation is involved in the regulation of RNA metabolism processes, including RNA capping, splicing, stabilization, translation and nuclear exportation

RNA methylations appear to function through nearly all steps of RNA metabolism, including RNA capping, splicing, stability, translation, and nuclear exportation (Fig. 3). The protein complex DXO/Dom3Z can decap the unmethylated cap and degrade the remaining part of pre-RNA [66]. Depletion of the m6A writer WTAP leads to abnormal mRNA isoforms [26]. It has been reported that m6A is enriched in exonic regions flanking 5′- and 3′-splice sites, where are the binding regions of mRNA splicing regulatory serine/arginine-rich protein 2 (SRSF2). Knockdown of the m6A eraser FTO results in elevated m6A level in the exonic regions, and enhanced mRNA binding ability of SRSF2, which lead to an increased inclusion of target exons [67]. These results indicate that RNA methylation may be involved in mRNA capping and splicing.

Nuclear export is an essential step for mRNA to ensure its translation in the cytoplasm. Knockdown of the m6A easer ALKBH5 induces poly (A)^+^ RNA retention in the nucleus [29]. The m5C reader ALYREF depletion also increases the nuclear retention of m5C-decorated mRNA [57]. These studies suggest that RNA methylation plays an important role in RNA nuclear export.

The dynamic synthesis and degradation of mRNA maintains an acute amount of mRNA in different contexts. For example, METTL3 knockdown decreases the m6A modification of cytokine signaling 2 (SOCS2) mRNA, leading to its degradation [68]. In addition, YTHDF2-bound mRNAs will translocate from translatable pool to mRNA decay sites (processing bodies) [12]. ALKBH1 (an m1A eraser) depletion increases the cellular level of tRNA^iMet^, suggesting that m1A modification is important for tRNA stability [53]. These data provide a strong connection between RNA methylation and mRNA stability. The m6A reader YTHDF1 has been found to interact with ribosomes and initiation factors, and increase cellular translational output, which is a piece of evidence for m6A function in translational regulation [69]. NSUN2-mediated m5C methylation inhibits the translation of the cyclin-dependent kinase inhibitor p27KIP1 [70]. Furthermore, recent ribosome profiling analyses suggest that both m6Am and m1A play important roles in regulating translation [37, 48].

### The roles of RNA methylations in biological processes

Considering the diverse regulation of RNA methylation on RNA fate, it would not be surprising that RNA methylation regulates many cellular and developmental processes. m6A modification has been documented to be important for nervous system development. *Mettl3* conditional knockout mice display the enhanced apoptosis of newborn cerebellar granule cells in the external granular layer, and lower levels of m6A modification in brain mRNA with aberrant RNA splicing and stability [71]. Postnatal deletion of *Mettl3* in the mouse hippocampus exhibit no significant morphological change but reduced memory consolidation ability [72]. Embryonic cortical radial glia cells in mice *deleted of Mettl3* or *Mettl4* manifests a prolonged cell cycle [73]. Additionally, FTO deficiency in mice decreases the proliferation and neuronal differentiation of adult neural stem cells, consequently leading to impaired learning and memory [74].

m6A modification is also important for germline development in mice. The ablation of mouse METTL3 inhibits spermatogonial differentiation and blocks the initiation of meiosis in germ cells [75]. The deficiency of ALKBH5 shows apoptosis of spermatocytes in pachytene and metaphase-stage, resulting in aberrant spermiogenesis and low number and poor quality of spermatozoa in the male mice [29]. YTHDC2 is highly expressed in mouse testes, and the spermatocytes of *Ythdc2* knockout mice fail to undergo pachytene, leading to defects in spermatocyte development [76].

RNA methylation also influences other fundamental physiological processes in development. Deletion of drosophila MTEEL3 homolog *Ime4* abolishes the m6A modification of the sex determination factor sex-lethal (*Sxl*), leading to X inactivation failure and improper sex determination [11, 77]. Zebrafish embryos that lack Mettl3 show a decrease in hematopoietic stem/progenitor cell numbers and defects in endothelial-to-hematopoietic transition [78]. Ythdf2-deficiency zebrafish embryos display developmental delay because of impaired decay of m6A-modified maternal RNAs during the maternal-to-zygotic transition [79].

## Roles of m6A in GI cancers

As mentioned above, RNA methylation has been implicated in diverse biological processes. There is no doubt that its dysregulation is highly associated with tumor initiation and progression. Accumulating evidence has shown that dysregulation of RNA methylation in cancer development, and the most well-studied one is m6A. m6A involves in cancer progression often through the actions of writers and easers that alter the abundance of m6A in the mRNA of oncogenes or tumor-suppressive genes and then the readers that recognize the modified sites to further upregulate oncogene or downregulate tumor-suppressive gene expression.

Many studies have shown that m6A modification plays a crucial role in various cancers. For example, the master methylase of m6A METTL3 has been reported to involve in many steps of RNA processing and the oncogenic pathway in different cancer types. In glioblastoma, METTL3 facilitates the maintenance and radiation resistance of glioma stem-like cells via increasing the m6A modification of *SRY-box transcription factor 2* (*SOX2*), which enhances the stability and protein expression of *SOX2* mRNA [80]. In non-small cell lung cancer, METTL3 triggers treatment resistance and metastasis of lung cancer cells by promoting the translation efficiency and stability of *yes-associated protein* mRNA [81]. METTL3 is also overexpressed in melanoma, which enhances the invasion and migration of human melanoma cells through increasing the expression of *matrix metallopeptidase 2* [82]. Meanwhile, many other RNA modifying proteins are also reported to be contributed to the initiation and progression of human cancers. Herein, we mainly focus on the biological roles of aberrant m6A modification in gastrointestinal cancers, including gastric cancer, colorectal cancer, liver cancer and pancreatic cancer (Table 1).

**Table 1:** RNA modifying proteins of m6A in gastrointestinal cancers

Cancer types	RMPs	Expression	Biological FUNCTIONS	Targets	References
Gastric cancer (GC)	METTL3	Up	Promote GC cell proliferation and liver metastasis	HDGF mRNA	[86]
			Facilitate the EMT program and matastasis	ZMYM1 mRNA	[87, 88]
	ALKBH5	Up	Promote GC cell invasion and migration	NEAT1	[89]
Colorectal cancer (CRC)	METTL3	Up	Promote CRC cell self-renewal and colorectal tumor growth and metastasis	SOX2 mRNA	[91]
			Promote CRC metastasis	pri-miR-1246	[91]
		Down	Inhibit CRC cell proliferation, invasion and migration	Unknown	[92]
			Inhibit CRC cell growth and invasion, tumor growth and metastasis	SOX4 mRNA	[94]
			Inhibit CRC cell proliferation and invasion, tumorigenicity and metastasis	XIST mRNA	[95]
	YTHDF1	Up	Promote CRC cell proliferation, drug sensitivity and tumor growth	Unknown	[96, 97]
Liver cancer (HCC)	METTL3	Up	Promote HCC cell proliferation, tumorigenicity and lung metastasis	SOCS2 mRNA	[68]
	METTL14	Down	Inhibit tumor metastasis	microRNA 126	[68, 98]
				DGCR8 mRNA	[99]
	WTAP	Up	Enhance HCC cell proliferation and tumor progression	ETS1 mRNA	[99]
	KIAA1429	Up	Promote the proliferation and invasion of HCC cells	ID2 mRNA	[100]
	FTO	Up	Promote the proliferation and tumor growth	PKM2 mRNA	[101]
		Down	Increase the apoptosis of ICC cells and reduce resistance to cisplatin treatment	Unknown	[102]
	YTHDF2	Down	Enhance inflammation, vascular reconstruction and metastatic progression of HCC cells	IL-11, SERPINE2 mRNA	[103]
Pancreatic cancer (PC)	METTL3	Up	Promote PC cell proliferation and invasion	Unknown	[105, 106]
	ALKBH5	Down	Inhibit PC cell motility	KCNK15-AS1	[107]
			Inhibit PDAC cell proliferation, migration, and invasion both in vitro and in vivo	WIF-1mRNA	[108]

### Gastric cancer

Gastric cancer develops from the lining of the stomach. More than 950,000 patients are diagnosed as gastric cancer, and about 720,000 patients died from gastric cancer every year worldwide [83]. It is usually divided into intestinal, diffuse and mixed histological subtypes according to Lauren classification [84]. METTL3 has been reported as an important player in gastric cancer development. Wang et al. [85] reported that the levels of m6A RNA and METTL3 were increased in gastric cancer tissues. METTL3, which expression is correlated with poor prognosis, promotes gastric cancer cell proliferation and liver metastasis by stimulating m6A modification of heparin binding growth factor (HDGF) mRNA. In addition, the m6A reader IGF2BP3 recognizes and binds to HDGF mRNA to enhance its stability. The increased HDGF then activates GLUT4 and ENO2 to induce glycolysis in gastric cancer cells. Yue et al. showed that METTL3 was upregulated in gastric cancer tissues, and was critical for epithelial-mesenchymal transition (EMT) process of gastric cancer cells and tumor metastasis [86]. Zinc finger MYM-type containing 1 (ZMYM1) mRNA is methylated by METTL3 and interacts with the m6A reader protein HuR to increase its stabilization. ZMYM1 binds to and represses the promoter of E-cadherin and then facilitates EMT program and the consequent metastasis of gastric cancer cells. Moreover, another two groups also found that METTL3 promoted the proliferation and mobility of gastric cancer cells [87, 88]. Zhang et al. [89] revealed that ALKBH5 promoted gastric cancer cell invasion by decreasing the methylation of the lncRNA nuclear paraspeckle assembly transcript 1 (NEAT1).

### Colorectal cancer

Colorectal cancer develops from one or a few small polyps inside the colon, and gradually develops into mature colorectal cancers. More than 1.8 million new colorectal cancer cases and 881,000 deaths are estimated to occur in 2018 [1]. Analyses of the Cancer Genome Atlas (TCGA) datasets show that the highly expression of *METTL3* mRNA in the metastatic colorectal cancer tissue is positively associated with poor prognosis of patients [90]. Knockdown of METTL3 suppresses the self-renewal, stem cell frequency and migration of colorectal cancer cells in vitro, and inhibits the colorectal tumor growth and metastasis in vivo. Mechanistically, METTL3 targets to SRY (sex determining region Y)-box 2 (SOX2) transcripts. The methylated SOX2 mRNAs are recognized and stabilized by the m6A reader IGF2BP2. Peng et al. [91] also reported that upregulated METTL3 facilitated colorectal cancer metastasis by methylation of pri-miR-1246, further influencing the downstream miR-1246/SPRED2/MAPK signaling axis. Deng et al. [92] found that knockdown of METTL3 promoted colorectal cancer cell proliferation and invasion possibly by increasing the phosphorylation of p38 and ERK. Interestingly, a strong correlation between METTL3 and ^18^F-FDG uptake is observed in colorectal cancer patients [93]. Furthermore, METTL3 modified m6A in HK2 and SLC2A1 transcripts to stabilize these two genes and activate the glycolysis pathway. m6A-mediated HK2 and SLC2A1 (GLUT1) stabilization depends on the reader protein IGF2BP2 or IGF2BP2/3, respectively. METTL14 expression is significant decreased in colorectal cancer and its lower expression is correlated with poor overall survival of patients [93]. Functionally, METTL14 knockdown leads to enhanced colorectal cancer cell growth and invasion in vitro and tumor growth and metastasis in vivo [94]. Mechanistically, METTL14 depletion results in decreased m6A modification and elevated expression of SRY-related high-mobility-group box 4 (SOX4) mRNA in a YTHDF2-dependent manner. Additionally, Zheng’s group also found that loss of METTL14 is associated with poor prognosis of colorectal cancer patients [94]. Knockdown of METTL14 promotes proliferation and invasion of colorectal cancer cells in vitro and enhanced tumorigenicity and metastasis in vivo. Furthermore, METTL14 depletion abolishes m6A level of *XIST* and attenuates its expression. YTHDF2 could recognized the m6A-methylated *XIST* to mediate its degradation [95]. Additionally, the m6A reader YTHDF1 was also found to be upregulated in colorectal cancer and correlated with poor prognosis of patients [96]. Furthermore, knockdown of YTHDF1 inhibits colorectal cancer cell proliferation, colonosphere formation, anti-cancer drug sensitivity and murine xenograft tumor growth in mice [97].

### Liver cancer

Liver cancer is predicted to be the fourth leading cause of cancer death worldwide, with about 841,000 new cases and 782,000 deaths annually [1]. The most common type of liver cancer is hepatocellular carcinoma (HCC), which is developed from the transformed hepatocytes and accounts for more than 75% of liver cancer cases [2]. The level of METTL3 mRNA is highly expressed in HCC, and its upregulation is correlated with poor prognosis of patients [68]. METTL3 has been further documented to not only induce HCC cell proliferation, colony formation and migration in vitro, but also promotes HCC tumorigenicity and lung metastasis by stabilizing suppressor of SOCS2 mRNA in a YTHDF2-dependent pathway. Unlike the significant increase of *METTL3*, METTL14 expression in HCC tissues shows a mild increase, and promotes HCC cell proliferation, colony formation and migration in vitro [68]. In contrast, Ma et al. [98] reported that METTL14 was downregulated in HCC tissues, and knockdown of *METTL14* enhances tumor metastasis. METTL14 was further showed to bind to the microprocessor protein DGCR8 mRNA and increase microRNA-126 in an m6A-dependent manner. WTAP also enhances the capability of HCC cell proliferation and tumor progression via the HuR-ETS1-p21/p27 axis [99]. Moverover, Cheng et al. [100] showed that another m6A writer KIAA1429 was upregulated in HCC tissues, and it promoted the proliferation and invasion of HepG2 cells by stabilizing the inhibitor of DNA binding 2 (ID2) mRNA. The m6A easer FTO is upregulated in HCC tissues, which correlates with poor prognosis of HCC patients [101]. FTO facilitates the proliferation and in vivo tumor growth via demethylating of *PKM2* mRNA and enhancing its translation efficiency. In contrast, FTO is found to be downregulated in intrahepatic cholangiocarcinoma (ICC). Loss of FTO in ICC is associated with tumor aggressiveness and poor prognosis. Suppression of FTO reduces the apoptosis of ICC cells and increases resistance to cisplatin treatment [102]. The expression of m6A reader YTHDF2 is decreased in HCC tissues and its downregulation is correlated with poor classification and prognosis of HCC patients [103]. YTHDF2 processes the decay of m6A-containing interleukin 11 and serpin family E member 2 transcripts to attenuate inflammation, vascular reconstruction and metastatic progression of HCC cells.

### Pancreatic cancer

Pancreatic cancer is the seventh leading cause of cancer death, with over 1,000,000 new cases and 65,000 deaths estimated globally [1]. Pancreatic ductal adenocarcinoma (PDAC) is the most lethal subtype of pancreatic cancer and accounts for more than 80% of pancreatic cancer patients [104]. METTL3 has been found to be highly expressed in pancreatic tumor tissues. METTL3-depleted pancreatic cancer cells display a decreased ability of cell proliferation and invasion, and an increased sensitivity to the treatment of anticancer reagents and irradiation [105, 106]. Recent study showed that ALKBH5 was downregulated in pancreatic cancer cells, and involved in the cell motility by demethylating the lncRNA *KCNK15-AS1* [107]. In addition, Shimamoto’s group showed that decreased ALKBH5 levels predicts poor clinical outcome of patients with pancreatic ductal adenocarcinoma (PDAC) [108]. Silencing ALKBH5 increases PDAC cell proliferation, migration, and invasion both in vitro and in vivo. Mechanistically, ALKBH5 suppresses Wnt signaling and its downstream targets though demethylation of m6A-modified WIF-1 transcripts.

## Targeting RNA modifying proteins for cancer treatment

Given that m6A modifying proteins play important roles in so many malignant behaviors of cancer cell, they could be used as potential therapeutic targets for cancer treatment. Some FTO inhibitors have been developed by different research groups to increase the m6A abundance [109, 110]. MA2, CS1 and CS2 have been shown to inhibit the growth and self-renewal of glioblastoma stem cells and leukemia stem cells, respectively [111, 112]. Another two promising FTO inhibitors, namely FB23 and FB23-2, which directly bind to FTO and selectively suppress the m6A demethylase activity of FTO, dramatically enhance the apoptosis of acute myeloid leukemia (AML) cells [113]. Peng et al. [114] showed another compound entacapone, a catechol-*O*-methyltransferase inhibitor, as an inhibitor of FTO. Furthermore, citrate and IOX3 may be potential inhibitors of another m6A demethylase, ALKBH5, which also maintains tumorigenicity of several types of cancer cells [115, 116]. In addition, the small molecule inhibitor BTYNB (2-{[(5-bromo-2-thienyl)methylene]amino} benzamide) has been demonstrated to show potently anti-proliferative effects in leukemia cells [117], ovarian cancer cell and melanoma cells by disrupting the reader protein IGF2BP1 [118]. Thus, researches on the functional mechanism of RNA modifying proteins will provide clues to the development of corresponding inhibitors for human cancer treatment. Moreover, studies focus on the investigation of RNA demethylase inhibitors are strongly needed in the near future.

## Conclusion and future perspectives

During the past 5 years, researchers have found that RNA modification are implicated in mRNA splicing, stability, translation, nuclear export and a wide range of biological processes. RNA modification and RNA-modifying proteins are often found to be aberrant expressed in tumor tissues. The changes of RNA modifications and their RNA-modifying proteins in cancer affects almost every aspect of RNA fate and is context-dependent. These RNA-modifying proteins linked to cancer are important regulators of cellular events by regulating RNA metabolism and expression of certain gene required for cancer cell proliferation, transformation, invasion, and other malignant behaviors. Studies on cancer epitranscriptomics focused on the complex context and heterogeneity of tumor could help us to better understand the exact roles of RNA modifications and their enzymes, which is benefit for exploring RNA modifying proteins as potential therapeutic targets.

Even though emerging data show the potential roles of RNA methylation in gastrointestinal cancer development, some important questions still remain to be addressed. First, how does the same RNA modifying protein selectively catalyze the different target transcripts in different cancer types. This may be attributed to the different upstream regulator of RNA modifying protein upon the specific cellular context in different cancers. Second, the inconsistent findings of the same RNA modifying protein were reported in the same cancer. One possible interpretation is that our current hypothesis is only based on individual cohorts with a relatively limited number of cancer patients with different genetic background and environmental exposure. Third, although a number of new types of RNA methylation in human have been identified until now, their functions in GI cancers need to be further investigated in the future.

## Data Availability

Not applicable.

## Abbreviations

ALYREF: Aly/REF export factor
Am: 2′-*O*-Methyladenosine
ALKBH5: AlkB homolog 5
EMT: Epithelial–mesenchymal transition
eRNAs: Enhancer RNAs
FTO: Fat mass and obesity-associated protein
GI: Gastrointestinal
Hm6A: *N*(6)-Hydroxymethyladenosine
HCC: Hepatocellular carcinoma
HDGF: Heparin binding growth factor
ID2: DNA binding 2
IGF2BP: Insulin-like growth factor 2 mRNA-binding proteins
m1A: *N*1-Methyladenosine
m5C: 5-Methylcytosine
m6A: *N*6-Methyladenosine
m6Am: 2-*O*-Dimethyladenosine
m7G: 7-Methyl guanosine
METTL3: Methyltransferase-like 3
METTL14: Methyltransferase-like 14
mRNAs: Messenger RNAs
NEAT1: Nuclear paraspeckle assembly transcript 1
PCIF1: Phosphorylated CTD interacting factor 1
PDAC: Pancreatic ductal adenocarcinoma
RMPs: RNA-modifying proteins
rRNAs: Ribosomal RNAs
SAM: *S*-Adenosyl-l-methionine
snRNAs: Small nuclear RNAs
snoRNAs: Small nucleolar RNAs
SOCS2: Cytokine signaling 2
SRSF2: Serine/arginine-rich protein 2
Sxl: Sex determination factor sex-lethal
SOX2: SRY (sex determining region Y)-box 2
tRNAs: Transfer RNAs
Tet: Ten–eleven translocation
TCGA: The Cancer Genome Atlas
WDR4: WD repeat domain 4
WTAP: Wilms tumor 1-associating protein
YBX1: Y-box binding protein 1
YTHDF: YTH-domain-containing family proteins
ZMYM1: Zinc finger MYM-type containing 1
